# Supplementary material for: Heterologous caffeic acid biosynthesis in Escherichia coli is affected by choice of tyrosine ammonia lyase and redox partners for bacterial Cytochrome P450
Source: Microb Cell Fact. 2020 Feb 11;19:26. doi: 10.1186/s12934-020-01300-9 (PMC7011507; doi:10.1186/s12934-020-01300-9)
Supplement: Supplementary file 1 — Additional file 1. DNA sequences of synthetic genes used in this study, two additional tables and two additional data figures. [file 12934_2020_1300_MOESM1_ESM.docx]

# Heterologous caffeic acid biosynthesis in Escherichia coli is affected by choice of tyrosine ammonia lyase and redox partners for bacterial Cytochrome P450

Kristina Haslinger^1^ and Kristala L.J. Prather^1^

^1^Department of Chemical Engineering, Massachusetts Institute of Technology, Cambridge, USA

Correspondence should be directed to [kljp@mit.edu](mailto:kljp@mit.edu)

## Supporting Information

### Sequences of codon optimized genes

FjTAL:

ATGAACACCATCAACGAATATCTGAGCCTGGAAGAATTTGAAGCCATTATCTTTGGCAATCAGAAAGTGACCATTAGTGATGTTGTTGTGAATCGCGTTAACGAGAGCTTTAACTTTCTGAAAGAATTTAGCGGCAACAAAGTGATCTATGGTGTGAATACCGGTTTTGGTCCGATGGCACAGTATCGTATTAAAGAAAGCGATCAGATTCAGCTGCAGTATAATCTGATTCGTAGCCATAGCAGCGGCACCGGTAAACCGCTGAGTCCGGTTTGTGCAAAAGCAGCAATTCTGGCACGTCTGAATACCCTGAGTCTGGGTAATAGCGGTGTTCATCCGAGCGTTATTAATCTGATGAGCGAACTGATCAACAAAGATATTACACCGCTGATTTTTGAACATGGTGGTGTTGGTGCAAGCGGTGATCTGGTTCAGCTGAGCCATCTGGCACTGGTTCTGATTGGTGAAGGTGAAGTTTTCTATAAAGGTGAACGTCGTCCGACACCGGAAGTTTTTGAAATTGAAGGTCTGAAACCGATCCAGGTGGAAATTCGCGAAGGTCTGGCCCTGATTAATGGCACCAGCGTTATGACCGGTATTGGTGTTGTTAATGTGTACCATGCAAAAAAACTGCTGGATTGGAGCCTGAAAAGCAGCTGTGCAATTAATGAACTGGTTCAGGCATATGATGATCACTTTAGCGCAGAACTGAATCAGACCAAACGTCATAAAGGTCAGCAAGAAATTGCACTGAAAATGCGTCAGAATCTGAGCGATAGCACCCTGATTCGCAAACGTGAAGATCATCTGTATAGCGGTGAAAACACCGAAGAAATCTTCAAAGAAAAAGTGCAAGAGTATTATAGCCTGCGTTGTGTTCCGCAGATTCTGGGTCCGGTTCTGGAAACCATTAACAATGTTGCAAGCATTCTGGAAGATGAATTTAACAGCGCAAACGATAACCCGATCATCGATGTTAAAAACCAGCATGTTTATCACGGTGGCAATTTTCATGGTGATTATATCAGCCTGGAAATGGATAAACTGAAAATCGTGATTACCAAACTGACCATGCTGGCAGAACGTCAGCTGAATTATCTGCTGAATAGCAAAATTAACGAACTGCTGCCTCCGTTTGTTAATCTGGGCACCCTGGGTTTTAACTTTGGTATGCAGGGTGTTCAGTTTACCGCAACCAGCACCACCGCAGAAAGCCAGATGCTGAGCAATCCGATGTATGTTCATAGCATTCCGAACAATAATGATAACCAGGATATTGTTAGCATGGGCACCAATAGCGCAGTTATTACCAGCAAAGTTATCGAAAATGCCTTTGAAGTTCTGGCCATTGAAATGATTACCATTGTTCAGGCGATTGATTATCTGGGCCAGAAAGATAAAATCAGCAGCGTTAGCAAAAAATGGTATGATGAAATCCGCAACATCATCCCGACCTTTAAAGAAGATCAGGTGATGTATCCGTTCGTGCAGAAAGTAAAAGACCACCTGATTAACAATtga

SeSam8:

ATGACCCAGGTTGTTGAACGTCAGGCAGATCGTCTGAGCAGCCGTGAATATCTGGCACGTGTTGTTCGTAGCGCAGGTTGGGATGCAGGTCTGACCAGCTGTACCGATGAAGAAATTGTTCGTATGGGTGCAAGCGCACGTACCATTGAAGAATATCTGAAAAGCGATAAACCGATCTATGGTCTGACCCAGGGTTTTGGTCCGCTGGTTCTGTTTGATGCAGATAGCGAACTGGAACAGGGTGGTAGCCTGATTAGCCATCTGGGCACCGGTCAGGGTGCACCGCTGGCACCGGAAGTTAGCCGTCTGATTCTGTGGCTGCGTATTCAGAATATGCGTAAAGGTTATAGCGCAGTTAGTCCGGTTTTTTGGCAGAAACTGGCAGATCTGTGGAATAAAGGTTTTACACCGGCAATTCCGCGTCATGGCACCGTTAGCGCAAGCGGTGATCTGCAGCCGCTGGCCCATGCAGCACTGGCATTTACCGGTGTTGGTGAAGCATGGACCCGTGATGCCGATGGTCGTTGGAGCACCGTTCCGGCAGTTGATGCACTGGCAGCCCTGGGTGCAGAACCGTTTGATTGGCCTGTTCGTGAAGCACTGGCCTTTGTTAATGGTACAGGTGCAAGCCTGGCAGTTGCAGTTCTGAATCATCGTAGTGCACTGCGTCTGGTTCGTGCATGTGCCGTTCTGAGCGCACGTCTGGCAACCCTGCTGGGTGCAAATCCGGAACATTATGATGTTGGTCATGGTGTTGCACGTGGTCAGGTTGGCCAGCTGACCGCAGCAGAATGGATTCGTCAGGGTCTGCCTCGTGGTATGGTTCGTGATGGTAGCCGTCCGCTGCAAGAACCGTATAGCCTGCGTTGTGCACCGCAGGTTCTGGGTGCGGTTCTGGATCAGCTGGATGGTGCCGGTGATGTTCTGGCACGCGAAGTTGATGGTTGTCAGGATAATCCGATTACCTATGAAGGTGAACTGCTGCACGGTGGTAATTTTCATGCAATGCCGGTTGGTTTTGCAAGCGATCAGATTGGTCTGGCAATGCATATGGCAGCATATCTGGCCGAACGTCAGCTGGGTCTGCTGGTTTCACCGGTTACCAATGGCGATCTGCCACCGATGCTGACACCGCGTGCAGGTCGTGGTGCAGGACTGGCAGGCGTTCAGATTAGCGCAACCAGCTTTGTTAGCCGTATTCGCCAGCTGGTTTTTCCGGCAAGCCTGACCACCCTGCCGACCAATGGTTGGAATCAGGATCATGTTCCGATGGCACTGAATGGTGCAAATAGCGTTTTTGAAGCCCTGGAACTGGGTTGGCTGACCGTGGGTAGCCTGGCCGTTGGTGTTGCCCAGCTGGCAGCAATGACAGGTCATGCAGCAGAAGGTGTTTGGGCTGAACTGGCAGGTATTTGTCCGCCTCTGGATGCCGATCGTCCACTGGGTGCCGAAGTTCGTGCAGCACGTGATCTGCTGAGTGCACATGCAGATCAGCTGCTGGTTGATGAAGCAGATGGTAAAGATTTTGGCtga

PCNA1-PdR:

ATGTTCAAAATCGTGTACCCGAACGCCAAAGATTTTTTCAGCTTTATTAACAGCATCACCAACGTGACCGATAGCATTATTCTGAACTTTACCGAAGATGGCATCTTTAGCCGTCATCTGACCGAAGATAAAGTTCTGATGGCAATTATGCGCATTCCGAAAGATGTTCTGAGCGAATATTCAATTGATAGCCCGACCAGCGTTAAACTGGATGTTAGCAGCGTGAAAAAAATCCTGAGCAAAGCAAGCAGCAAAAAAGCAACCATTGAACTGACCGAAACCGATAGCGGTCTGAAAATTATCATCCGTGATGAAAAAAGCGGTGCCAAAAGCACCATTTATATCAAAGCAGAAAAAGGCCAGGTTGAACAGCTGACAGAACCGAAAGTTAATCTGGCAGTGAATTTTACCACCGATGAAAGCGTTCTGAATGTTATTGCAGCAGATGTTACCCTGGTTGGTGAAGAAATGCGTATTAGCACCGAAGAGGACAAAATCAAAATTGAAGCCGGTGAAGAGGGTAAACGTTATGTTGCATTTCTGATGAAAGACAAGCCGCTGAAAGAACTGAGCATTGATACCAGCGCCAGCAGCAGCTATAGCGCAGAAATGTTTAAAGATGCAGTTAAAGGTCTGCGTGGTTTTAGCGCACCGACAATGGTGAGCTTTGGTGAAAATCTGCCGATGAAAATTGATGTTGAAGCAGTTAGCGGTGGCCACATGATTTTTTGGATTGCACCGCGTTTAGGTGGTGGTGGTAGCGGTGGTGGCGGTTCAATGAATGCAAATGATAATGTTGTTATCGTTGGCACCGGTCTGGCAGGCGTTGAAGTTGCATTTGGCCTGCGTGCAAGCGGTTGGGAAGGTAATATTCGTCTGGTGGGTGATGCAACCGTTATTCCGCATCATCTGCCTCCGCTGAGTAAAGCATATCTGGCAGGTAAAGCAACCGCAGAAAGCCTGTATCTGCGTACACCGGATGCCTATGCAGCACAGAATATTCAGCTGTTAGGTGGCACCCAGGTTACCGCAATTAATCGTGATCGTCAGCAGGTTATTCTGAGTGATGGTCGTGCACTGGATTATGATCGTCTGGTTCTGGCAACCGGTGGTCGTCCGCGTCCGCTGCCGGTTGCAAGTGGTGCAGTTGGTAAAGCCAATAACTTTCGTTATCTGCGCACCCTGGAAGATGCAGAATGTATTCGTCGTCAGCTGATTGCAGATAATCGCCTGGTTGTTATTGGTGGTGGCTATATTGGTCTGGAAGTTGCAGCAACCGCCATTAAAGCAAATATGCATGTGACCCTGCTGGATACCGCAGCACGTGTTCTGGAACGTGTTACCGCACCGCCTGTTAGCGCCTTTTATGAACATCTGCATCGTGAAGCGGGTGTTGATATTCGCACCGGTACACAGGTTTGTGGTTTTGAAATGAGCACCGATCAGCAGAAAGTTACCGCAGTTCTGTGTGAAGATGGTACACGTCTGCCTGCAGATCTGGTTATTGCCGGTATTGGCCTGATTCCGAATTGTGAACTGGCAAGCGCAGCAGGTCTGCAGGTTGATAATGGTATTGTTATTAACGAACACATGCAGACCAGCGATCCGCTGATTATGGCAGTTGGTGATTGTGCACGTTTTCATAGCCAGCTGTATGATCGTTGGGTTCGTATTGAAAGCGTGCCGAATGCACTGGAACAGGCACGTAAAATTGCAGCAATTCTGTGTGGCAAAGTTCCGCGTGATGAAGCAGCACCGTGGTTTTGGAGCGATCAGTATGAAATCGGCCTGAAAATGGTTGGTCTGAGTGAAGGTTATGATCGCATTATTGTTCGTGGTAGCCTGGCACAGCCGGATTTTTCAGTTTTTTATCTGCAGGGTGATCGTGTGCTGGCAGTTGATACCGTTAATCGTCCGGTTGAATTTAATCAGAGCAAGCAGATTATTACCGATCGTCTGCCGGTGGAACCGAACCTGCTGGGTGATGAAAGTGTTCCTCTGAAAGAAATTATTGCCGCAGCAAAAGCAGAACTGAGTAGCGCATAA

### Additional Tables

Additional file 1:Table S1: Comparison of select tyrosine ammonia lyase enzymes characterized in other studies.

| Enzyme | K_m_ [mM] | k_cat_ [s^-1^] | k_cat_/K_m_ [mM^-1^ s^-1^] | Ratio TAL:PAL activity | Reference |
| --- | --- | --- | --- | --- | --- |
| RgTAL (enzyme purified by anion exchange chromatography, ammonium sulfate precipitation and hydrophobic interaction chromatography; assay performed at pH 8.5; T=25°C) | 615 | 0.53 | 8.6×10^-4^ | 10 | [1] |
| RgTAL (enzyme purified by anion exchange chromatography, ammonium sulfate precipitation and hydrophobic interaction chromatography; assay performed at pH 9.5; T=25°C) | 67.7 | 0.93 | 1.37x10^-2^ | 10 | [1] |
| FjTAL (enzyme purified by metal affinity chromatography; assay performed at pH 9.5; T=30°C) | 6.7x10^-3^ | 0.023 | 2.99 | 2400 | [2] |
| SeSam8 (enzyme purified by metal affinity chromatography; assay performed at pH 9.5; T=30°C) | 4.7x10^-3^ | 0.015 | 3.05 | 1200 | [2] |
| RgTAL (enzyme purified by metal affinity chromatography; assay performed at pH 8.5; T=40°C) | 0.38 | 114 | 298 | n.d. | [3] |

Additional file 1: Table S2: Plasmids generated for this study including cloning strategies used.

| **plasmid name** | **Plasmid description**  **[backbone::MCSI_MCSII]*** | **cloning method** | **Primers (binding sequence in lowercase, overhang in uppercase letters)** | **source** |
| --- | --- | --- | --- | --- |
| IR54 | pKVS45::PdR-Pux operon | / | / | [4] |
| IR64 | pCDFduet::_6His-CYP199A2 F185L NΔ7 | / | / | [4] |
| c22 | pRSFduet::6His-RgTAL | Golden Gate Assembly enzyme: SapI template: IR66 | insert MCSI fwd CGTCAACGCTCTTCCtccgcgtccgacctcgca insert MCSI rev CGTCAACGCTCTTCCcttatgccagcattttcagcagc backbone fwd CGTCAACGCTCTTCCaagcggccgcataatgctta backbone rev CGTCAACGCTCTTCCggagccatttggcgcgccgagctcga | This study |
| c25 | pCDFduet::_PCNA3-CYP199A2 F185L NΔ7 | Golden Gate Assembly enzyme: BsaI templates: pHSG-PCNA3 and IR66 | PCNA3 fwd CGTCAACGGTCTCGACATatgatatatcttaaatcttttgaaaggaatataag PCNA3 rev CGTCAACGGTCTCGGCATagatccaccaacttttggagc CYP199A2 F185L NΔ7 fwd CGTCAACGGTCTCGatgccggttacgacgccg CYP199A2 F185L NΔ7 rev CGTCAACGGTCTCGGACAtcaggccggggtcagttg backbone fwd CGTCAACGGTCTCGtgtcttcggtaccctcgag backbone rev CGTCAACGGTCTCGatgtatatctccttcttatacttaactaa | This study |
| c28 | pETduet::6His-PCNA2-Pux_PCNA1-PdR(opt) | classic cloning  MCSII: NdeI/KpnI (insert: gblocks), backbone: c58 | insert MCS II fwd GATCTACATatgttcaaaatcgtgtacccgaa insert MCSII rev GATCTAggtaccactagtatttatgcgctac | This study |
| c50 | pETduet::6His-Pux_PdR(opt) | classic cloning  MCSI: SacI/NotI (template: IR54),  MCSII: NdeI/EcoRV (template: gblocks) | insert MCSI fwd GATCGAGCTCAatgcccagtatcacgttcatt insert MCSI rev GATCgcggccgcaagcttgtcg insert MCSII fwd GATCATCATatgaatgcaaatgataatgttgttatcgttg insert MCSII rev GATCTAGGTACCactagtatttatgcgctac | This study |
| c62 | pETduet::6His-Pux_PuR | classic cloning  MCSI: SacI/NotI (template: IR54),  MCSII: NdeI/KpnI (template: pACYCduet::PuR_Pux) | insert MCSI fwd GATCGAGCTCAatgcccagtatcacgttcatt insert MCSI rev GATCgcggccgcaagcttgtcg insert MCSII fwd GATCTACATatggacgacacggtcttgattg insert MCSII rev GATCGGTACCGAAGACAttacgccgccgccttcttc | This study |
| c63 | pETduet::6His-PCNA2-Pux_PCNA1-PuR | restriction digest/ligation, enzymes: MCS2, NdeI/KpnI (insert: c64), backbone: c28 | / | This study |
| c71 | pRSFduet::6His-FjTAL | classic cloning  enzymes: BamHI/ NotI (template: gblocks) | insert MCSI fwd GATCAggatccgagcagcggc insert MCSI rev GATCaagcggccgcaagctttca | This study |
| c72 | pRSFduet::6His-SeSam8 | classic cloning  enzymes: BamHI/ NotI (template: gblocks) | insert MCSI fwd GATCAggatccgagcagcggc insert MCSI rev GATCaagcggccgcaagctttca | This study |
| c84 | pCDFduet::6His-Pux_6His-CYP199A2F185L NΔ7 | MCSI: classic cloning, enzymes: SacI/NotI (template: c50); MCSII: restriction digest/ligation, NdeI/AvrII (insert: IR64) | insert MCSI fwd GATCGAGCTCAatgcccagtatcacgttcatt insert MCSI rev GATCgcggccgcaagcttgtcg | This study |
| c86 | pETduet::6His-Pdx_PdR (opt) | classic cloning  enzymes: MCSI, SacI/HindIII, template: *Pseudomonas putida* gDNA; backbone:c50 | insert MCSI fwd GATCATGAGCTCAatgtctaaagtagtgtatgtgtcacatg insert MCSI rev GATCATAAGCTTGTCGACttaccattgcctatcgggaacatc | This study |
| c88 | pETduet::6His-PCNA2-Pdx_PCNA1-PdR (opt) | classic cloning  enzymes: MCSI, BseRI/SalI, template: *Pseudomonas putida* gDNA; backbone: c28 | insert MCSI fwd GATCATCCTCCACCGCCTCCTCCACCGCCACCACCGCCGCCTCCAC CTCCACCGCCGCCCGGTatgtctaaagtagtgtatgtgtcacatg insert MCSI rev GATCATAAGCTTGTCGACttaccattgcctatcgggaacatc | This study |
| c96 | pCDFduet::_PCNA1-GGS-CYP199A2 F185L NΔ7 | Round-the-Horn PCR to shorten peptide linker (template:c77) | fwd cggttcaatgccggttacg rev cctaaacgcggtgcaatccaaa | This study |
| c97 | pETduet::6His-PCNA2-Pux_PCNA3-(GGGS)2-PdR (opt) | Round-the-Horn PCR to expand peptide linker (template: c80) | fwd AGCGGTGGTggtggatctatgaatgcaaatgataatgttg rev ACCACCACCACCaacttttggagctaataaataagtaactttccc | This study |
| c98 | pETduet::6His-PCNA2-Pux_PCNA3-(GGGS)2-PuR | Round-the-Horn PCR to expand peptide linker (template: c81) | fwd AGCGGTGGTggtggatctatggacgacacg rev ACCACCACCACCaacttttggagctaataaataagtaactttccc | This study |
| c106 | pETduet::6His-PCNA2-Pdx_PCNA3-GGS-PdR (opt) | restriction digest/ligation, enzymes: MCS2, NdeI/KpnI (insert: c97), backbone: c88 | / | This study |
| c185 | pRSFduet::6-His-FjTAL_Pux | Classic cloning of pux gene into c71: enzymes MCS2, NdeI/KpnI (insert: c62), backbone: c71 | fwd GATCATCATatgcccagtatcacgttcattc  rev GATCATGGTACCtcagacctgacgatccggaatc | This study |
| **PCR templates and additional plasmids** | | | | |
| IR66 | pCDFduet::RgTAL_6His-CYP199A2 F185L NΔ7 | / | / | [4] |
| c55 | pCDFDuet::_CYP199A2F185L NΔ7 (without His-Tag) | Golden Gate Assembly  enzyme: BsaI  template: IR66 | insert fwd CGTCAACGGTCTCGacatatgccggttacgacgccg insert rev CGTCAACGGTCTCGgacatcaggccggggtcagttg backbone fwd CGTCAACGGTCTCGacatatgccggttacgacgccg backbone rev CGTCAACGGTCTCGacatatgccggttacgacgccg | This study |
| c58 | pETduet::6His-PCNA2-Pux_ | MCSI: Golden Gate Assembly  enzyme: BsaI  templates: pHSG-PCNA2 (two-step PCR) and IR54 | PCNA2 fwd CGTCAACGGTCTCCCTCAatgaaagctaaggtaattgacgctg PCNA2 rev 1 CGTCAACGGTCTCCTGGCGGTGGAGGAGGCGGTGGAGGTGGAGGG CTGCCGCCACCgtctgcccttggtgcaatg PCNA2 rev 2 CGTCAACGGTCTCCTGGCGGTGGAGGAGGCGGTG Pux fwd CGTCAACGGTCTCCGCCACCACCGCCGCCTCCACCTCCACCGCCGCCCGGT atgcccagtatcacgttcattc Pux rev CGTCAACGGTCTCGCGACtcagacctgacgatccggaatc backbone fwd CGTCAACGGTCTCGgtcgacaagcttgcggccgc backbone rev CGTCAACGGTCTCGtgagctcgaattcggatcctggctgtggtga | This study |
| c64 | pETDuet::_PCNA1-PuR | Golden Gate Assembly  enzyme: BsaI  templates: c28 and pACYC::PuR_Pux | PCNA1 fwd CGTCAACGGTCTCGACATtttaagattgtttaccctaatgcaaaagac PCNA1 rev CGTCAACGGTCTCGactcccgccgccaccagaac PuR fwd CGTCAACGGTCTCGGAGTatggacgacacggtcttgattg PuR rev CGTCAACGGTCTCGGACAttacgccgccgccttcttc backbone fwd CGTCAACGGTCTCGtgtcttcggtaccctcgag backbone rev GGTAAACAATCTTAAAtgagctcgaattcggatcctgg | This study |
| c77 | pCDFduet::_PCNA1-(GGGS)2-CYP199A2 F185L NΔ7 | CPEC  template: c28, backbone: c55 | insert fwd tataagaaggagatatacatATGTTCAAAATCGTGTACCCG insert and CPEC rev GACGGCGTCGTAACCGGCATtgaaccgccaccaccgctac CPEC fwd GTAGCGGTGGTGGCGGTTCAatgccggttacgacgccgtc | This study |
| c78 | pETduet::6His-PCNA2-Pux_PdR | classic cloning, enzymes: MCSII NdeI/KpnI (template: c28, backbone: c28) | insert MCSII fwd GATCATCATatgaatgcaaatgataatgttgttatcgttg insert MCSII rev GATCTAGGTACcactagtatttatgcgctac | This study |
| c79 | pETduet::6His-PCNA2-Pux_PuR | classic cloning, enzymes: MCSII NdeI/KpnI (template: c62, backbone: c28) | insert MCSII fwd GATCTACATatggacgacacggtcttgattg insert MCSII rev GATCGGTACCGAAGACattacgccgccgccttcttc | This study |
| c80 | pETduet::6His-PCNA2-Pux_PCNA3-PdR (GGS) | CPEC  template: c25 , backbone: c78 | insert fwd AGTATAAGAAGGAGATATACATatgatatatcttaaatcttttgaaaggaatataag attga insert and CPEC rev ACATTATCATTTGCATTCATagatccaccaacttttggag CPEC fwd CTCCAAAAGTTGGTGGATCTatgaatgcaaatgataatgttgtt | This study |
| c81 | pETduet::6His-PCNA2-Pux_PCNA3-PuR (GGS) | CPEC  template: c25 , backbone: c79 | insert fwd AGTATAAGAAGGAGATATACATatgatatatcttaaatcttttgaaaggaatataag attga insert and CPEC rev ATCAAGACCGTGTCGTCCATagatccaccaacttttggag CPEC fwd CTCCAAAAGTTGGTGGATCTatggacgacacggtcttgat | This study |
| pACYCduet::PuR_Pux | pACYCduet::PuR_Pux | / | / | Dr. Stephen G. Bell |
| pHSG-PCNA2 | pHSG-PCNA2 | / | / | [5] |
| pHSG-PCNA3 | pHSG-PCNA3 | / | / | [5] |

*MCS: multiple cloning site (each with its own T7 promoter and terminator)

### Additional Figures


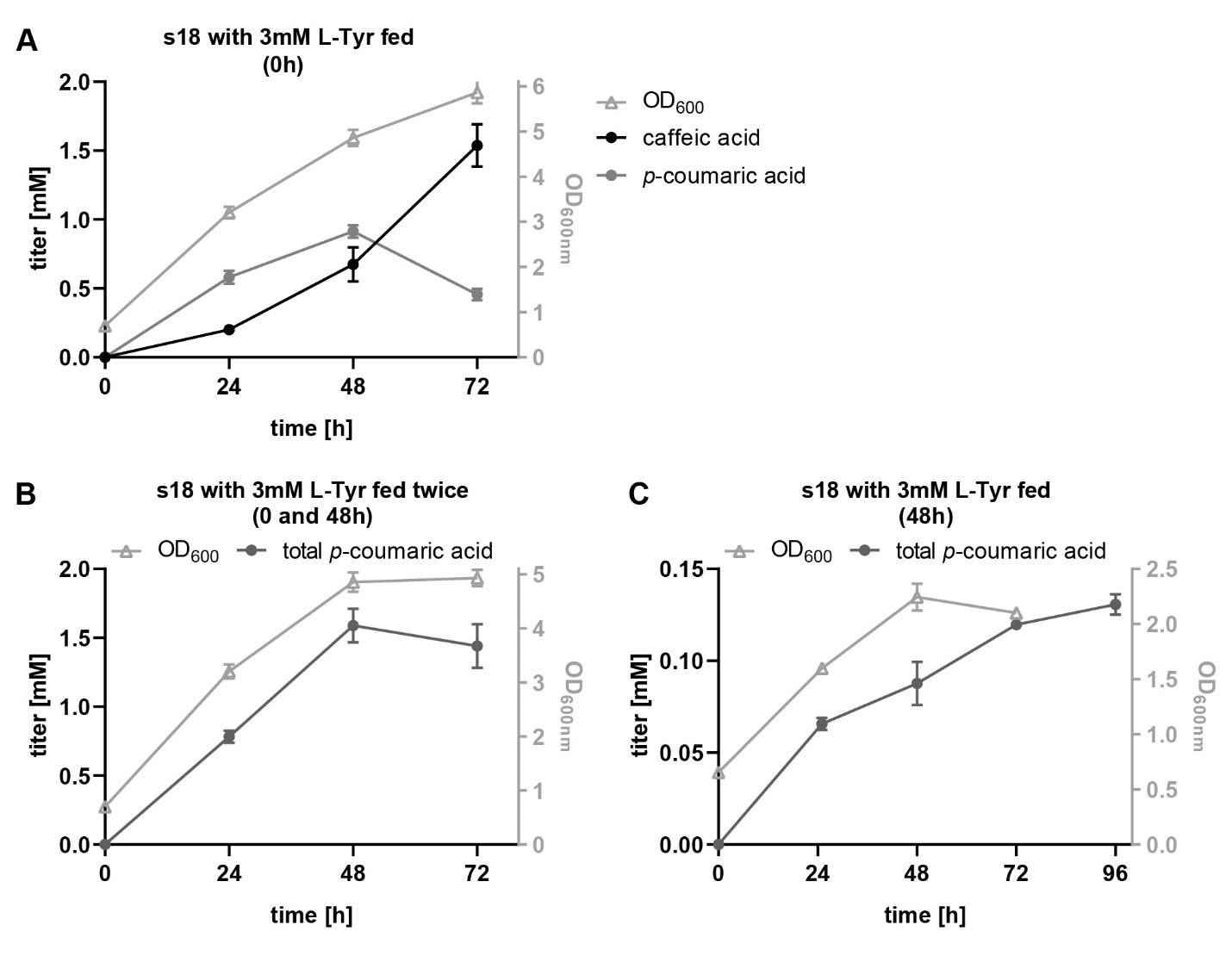


Additional file 1: Figure S1: Time course experiments with strain s18 expressing the Pux/PuR redox system with a doubled gene dose of pux under different L-tyrosine feeding strategies and 4% (w/v) of glucose. Feeding of 3 mM L-tyrosine at t=0h p.i. (A), at t=0h p.i. and t=48h p.i. (B) and at t=48h p.i.(C). Total p-coumaric acid titers plotted in panels B and C were calculated by summing up the measured titers of p-coumaric and caffeic acid. In these experiments, no distinct increase in titers was observed after the addition of L-tyrosine at 48h p.i.. This indicates that the additional tyrosine could not be converted to coumaric or caffeic acid, most likely because the tyrosine ammonia lyase enzyme was inactive or denatured. (error bars=standard deviation of biological replicates, n=3).

Additional file 1: Figure S2*:* Stacked histograms of *p-*coumaric and caffeic acid titers after 96 h of fermentation with glucose as the only carbon source for select strains expressing the two-step pathway with varying numbers of gene copies of *pux*. (error bars=standard deviation of biological replicates, n=3).

## References

1. Xue Z, McCluskey M, Cantera K, Ben-Bassat A, Sariaslani FS, Huang L. Improved production of p-hydroxycinnamic acid from tyrosine using a novel thermostable phenylalanine/tyrosine ammonia lyase enzyme. Enzyme Microb Technol. 2007;42:58–64.

2. Jendresen CB, Stahlhut SG, Li M, Gaspar P, Siedler S, Förster J, et al. Novel highly active and specific tyrosine ammonia-lyases from diverse origins enable enhanced production of aromatic compounds in bacteria and yeast. Appl Environ Microbiol. 2015; April. doi:10.1128/AEM.00405-15.

3. Zhou S, Liu P, Chen J, Du G, Li H, Zhou J. Characterization of mutants of a tyrosine ammonia-lyase from *Rhodotorula glutinis*. Appl Microbiol Biotechnol. 2016;100:10443–52. doi:10.1007/s00253-016-7672-8.

4. Rodrigues JL, Araújo RG, Prather KLJ, Kluskens LD, Rodrigues LR. Heterologous production of caffeic acid from tyrosine in *Escherichia coli*. Enzyme Microb Technol. 2015;71:36–44. doi:10.1016/j.enzmictec.2015.01.001.

5. Hirakawa H, Nagamune T. Molecular assembly of P450 with ferredoxin and ferredoxin reductase by fusion to PCNA. ChemBioChem. 2010;11:1517–20.
